# Acetylation of histone H4K4 is cell cycle regulated and mediated by HAT3 in *Trypanosoma brucei*

**DOI:** 10.1111/j.1365-2958.2007.06079.x

**Published:** 2008-02

**Authors:** T Nicolai Siegel, Taemi Kawahara, Jeffrey A DeGrasse, Christian J Janzen, David Horn, George A M Cross

**Affiliations:** 1 Laboratory of Molecular Parasitology, The Rockefeller University New York, USA; 2 London School of Hygiene and Tropical Medicine London, UK; 3 Laboratory for Mass Spectrometry and Gaseous Ion Chemistry, The Rockefeller University New York, USA

## Abstract

Post-translational histone modifications have been studied intensively in several eukaryotes. It has been proposed that these modifications constitute a ‘histone code’ that specifies epigenetic information for transcription regulation. With a limited number of histone-modifying enzymes, implying less redundancy, *Trypanosoma brucei* represents an excellent system in which to investigate the function of individual histone modifications and histone-modifying enzymes. In this study, we characterized the acetylation of lysine 4 of histone H4 (H4K4), the most abundant acetylation site in *T. brucei* histones. Because of the large sequence divergence of *T. brucei* histones, we generated highly specific antibodies to acetylated and unmodified H4K4. Immunofluorescence microscopy and Western blots with sorted cells revealed a strong enrichment of unmodified H4K4 in S phase and suggested a G1/G0-specific masking of the site, owing to non-covalently binding factors. Finally, we showed that histone acetyltransferase 3 (HAT3) is responsible for H4K4 acetylation and that treatment of cells with the protein synthesis inhibitor cycloheximide led to an almost instantaneous loss of unmodified H4K4 sites. As HAT3 is located inside the nucleus, our findings suggest that newly synthesized histone H4 with an unmodified K4 is imported rapidly into the nucleus, where it is acetylated, possibly irreversibly.

## Introduction

Histone post-translational modifications (PTMs) play essential roles in chromatin assembly, replication, recombination, DNA damage repair, transcriptional regulation and perpetuation of epigenetic information (reviewed in Fischle *et al*., 2003). Among the many identified PTMs, acetylations, methylations and phosphorylations are the best characterized, and many enzymes responsible for the addition and removal of these modifications have been identified. It has been proposed that histone PTMs constitute a so-called ‘histone-code’ (Strahl and Allis, 2000) of epigenetic information for transcription regulation and chromatin structure. Deciphering this code in higher eukaryotes has been complicated by the large number of histone PTMs and histone-modifying enzymes and a high degree of redundancy. Research on lower eukaryotes with a more concise chromatin composition might more easily reveal the critical functions of individual histone PTMs. One such eukaryote, *Tetrahymena thermophila*, is the subject of intense study. This ciliated protozoan contains two nuclei: a transcriptionally inactive germline micronucleus and a large polyploid somatic macronucleus that is transcriptionally active. *Tetrahymena* has been an invaluable model for understanding chromatin structure and function, despite its seemingly unorthodox genetics. Its transcriptionally active macronuclei served as a source for hyperacetylated histones and for the purification of the first histone acetyltransferase (HAT) (Brownell *et al*., 1996).

To fully understand the complex network of histone modifying enzymes, histone PTMs and factors binding to these modifications, it will be important to look towards other lower eukaryotes and to take advantage of their less complex histone-modifying machinery. The functions of histone PTMs in *Trypanosoma brucei*, the causative agent of African sleeping sickness, has been studied only marginally, even though *T. brucei* possesses many attributes of a successful model organism. It can be cultured readily in liquid media or in small rodents. Genetic manipulation is straightforward, RNAi can be used to efficiently deplete essential proteins and its genome has been sequenced (Berriman *et al*., 2005). Furthermore, epigenetic mechanisms seem to regulate the mono-allelic expression of genes encoding the variant surface glycoprotein that constitutes the surface coat of the mammalian-infective bloodstream form (BF) (reviewed in Cross *et al*., 1998; Pays *et al*., 2004). The variant surface glycoprotein is transcribed from 1 of ∼20 polycistronic transcription units known as expression sites, which are located adjacent to telomeres. Upon ingestion of BF by the tsetse vector and differentiation into procyclic forms (PFs), all expression sites are silenced. Again, chromatin remodelling appears to play a role in this developmental expression (reviewed in Sullivan *et al*., 2006).

Despite its early divergence from other eukaryotes, patterns of histone PTM seem to be conserved in trypanosomes. For example, methylation of histone H3 by homologues of Dot1 has been studied in *T. brucei* (Janzen *et al*. 2006a). In addition, extensive acetylation of the N-terminal tail of histone H4 has been observed in *T. brucei* and *T. cruzi* (da Cunha *et al*., 2006; Janzen *et al*., 2006b). In this study, we characterize H4K4, the most commonly acetylated [∼80% in *T. brucei* (Janzen *et al*., 2006b)] site in trypanosome histones, and possibly the equivalent site to H4K5 in other eukaryotes, whose acetylation plays a role in histone deposition (Sobel *et al*., 1995), cell cycle progression (Megee *et al*., 1990), transcription activation (Schiltz *et al*., 1999) and DNA damage repair (Bird *et al.*, 2002). Several HATs and histone deacetylases (HDACs) modulate H4K5 acetylation (Parthun *et al*., 1996; Rundlett *et al*., 1996).

## Results

### Generation of specific antibodies

**Fig. 1:**
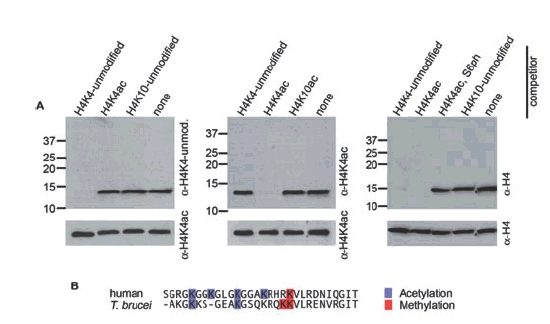
Characterization of antibodies. A. Western blot of whole-trypanosome extracts (2 × 10^6^ cells lane^−1^) using α-H4K4-unmodified, α-H4K4ac or general α-H4. Peptide competitors used are shown above. To confirm equal loading, blots were stripped and reprobed with α-H4K4ac or α-H4 without peptide competitors. Additional data are presented in Fig. S1. B. Sequences of N-terminal tails of human and trypanosome histone H4.

The large differences in *T. brucei* histone sequences (human and *T. brucei* H4 N-terminal sequences are compared in Fig. 1B) prohibits the use of commercially available antibodies to specific modifications. Thus, it was necessary to generate antibodies to both the acetylated and unmodified H4K4. A third antibody that recognized the N-terminal tail of histone H4 regardless of the acetylation state of H4K4 was fortuitously obtained after immunization with the same peptide that had been used to raise α-H4K4-unmodified. This antibody was therefore considered a general H4 antibody. Antibody specificity was tested by pre-incubation with peptide competitors before Western blotting (Fig. 1A) or immunofluorescence (IF) analysis (data not shown). The H4K4-unmodified and H4K4ac antibodies showed affinity only for their corresponding peptides. No cross-reactivity to other modified or unmodified sites could be detected. The general histone H4 antibody, on the other hand, showed high affinity for both the unmodified and to the acetylated peptide, but did not react with other histones.

### H4K4 epitope masking in cells during G1/G0

**Fig. 2:**
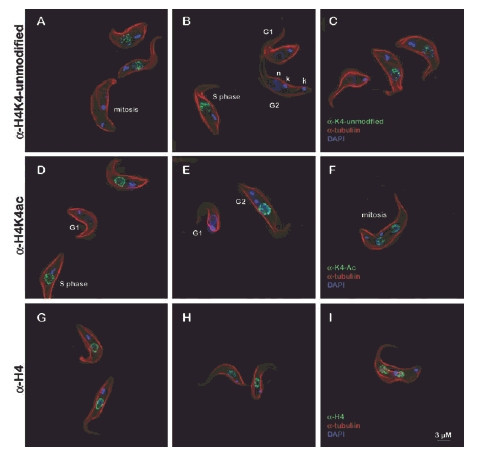
Cell cycle-dependent acetylation of H4K4. A–C. Unmodified H4K4 is only detectable in cells with an elongated kinetoplast (k), denoting S phase. Cells in G1/G0 contain one nucleus and one kinetoplast (1N1K). Cells in G2/M contain one nucleus and two kinetoplast (1N2K). D–F. The H4K4ac site is detectable in all cells except G1 cell (1N1K). G–I. α-H4 binds to histones in all cells.

Immunofluorescence analysis using DeltaVision deconvolution microscopy and the newly generated histone antibodies revealed a punctate pattern throughout the nucleus, excluding only the nucleolus (Fig. 2). A punctate pattern has generally been observed in other organisms, when using antibodies against histone PTMs.

Although antibodies to both the unmodified and acetylated H4K4 worked for IF, neither antibody reacted with cells in G1/G0 (Fig. 2D–F). In contrast, the general H4 antibody bound throughout the cell cycle (Fig. 2G–I), suggesting that the H4K4 site may have been specifically blocked in G1/G0, either by another covalent modification close to H4K4 or by a factor binding to that site. Sites for potential covalent modification are present at K2, K5 and S6, but no modifications have been detected at these sites in PF (Janzen *et al*., 2006b). Only very minor (< 10%) levels of acetylation have been detected at K2 and K5 in BF (Mandava *et al*., 2007), ruling out the possibility that these modifications could completely block binding of antibody to H4K4 throughout G1/G0. Neither study investigated the possible phosphorylation of H4S6. To test whether phosphorylation of H4S6 could explain our observations, we generated a synthetic doubly modified phosphoacetylated peptide (K4ac, S6ph). Peptide competition experiments demonstrated that the general H4 antibody did not bind the phosphorylated peptide (Fig. 1A). As we know from IF studies that the general H4 antibody binds to cells in G1/G0, we concluded that phosphorylation of H4S6 is not responsible for blocking the H4K4 site during G1/G0. We therefore investigated the possibility of blockage by non-covalent interactions.

**Fig. 3:**
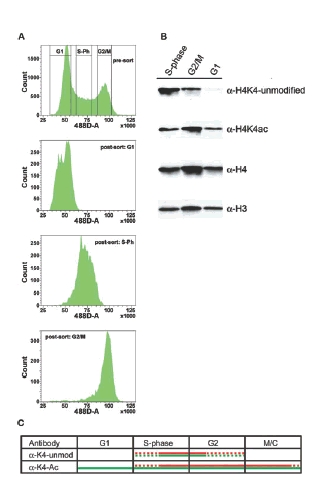
α-H4K4ac binds to cells in G1/G0 under denaturing conditions. A. Unfixed DyeCyclin Orange-stained cells were sorted based on DNA content and analysed by Western blotting. The membrane was stripped repeatedly and reprobed with the listed antibodies. B. FACS analysis of pre- and post-sorted cells. C. Diagram summarizing the detection of the H4K4-unmodified and H4K4ac marks as determined by IF (red line) and Western blot (green line) analysis. A dashed line indicates a possible presence.

If H4K4 were masked by a non-covalently interacting factor *in vivo*, we reasoned that SDS-PAGE would disrupt the interaction and the H4K4 site in cells in G1/G0 would become accessible to the antibodies. It is not possible to synchronize the cell cycle in *T. brucei*, so cells were sorted according to their DNA content. To avoid potential problems reversing cross-links arising from fixation, we sorted unfixed DyeCyclin Orange-stained cells and then analysed them by Western blot. Under these denaturing conditions, the H4K4ac antibody bound to H4 from cells in G1/G0 (Fig. 3A), suggesting that masking of H4K4 in the IF analysis was indeed caused by some non-covalently binding factor (Fig. 3C). Prior to Western blot analysis, all fractions were re-sorted to confirm that the initial sort had generated homogenous cell populations (Fig. 3B).

### Unmodified H4K4 is strongly enriched in S phase cells

Western blot analysis of cells sorted for DNA content also revealed a strong enrichment of unmodified H4K4 in cells in S phase (Fig. 3A). In IF, only cells with an elongated kinetoplast showed a signal with the unmodified H4K4 antibody (Fig. 2A–C). PFs with an elongated kinetoplast had originally been classified as cells in G2 (Woodward and Gull, 1990). However, the cells used by Woodward and Gull had a population doubling time of 8.5 h compared with 12.5 h in the present experiments. Consequently, we reinvestigated the timing of kinetoplast division in relationship to the cell cycle and found that kinetoplast division, visualized by an elongated kinetoplast, is initiated during nuclear S phase (T.N. Siegel, D.R. Hekstra and G.A.M. Cross, in preparation). We concluded, therefore based on our IF analysis, that the unmodified H4K4 site is strongly enriched in cells in S phase. Epitope masking during other cell cycle stages can be excluded as both our IF and Western blot data show a strong enrichment of unmodified H4K4 in cells in S phase (Figs 2A–C and Figs 3B–C).

### HAT3 is responsible for acetylation of H4K4

**Fig. 4:**
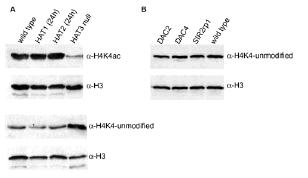
HAT3 acetylates H4K4. Western blot analysis of the H4K4 acetylation state in BF cell lysates. A. HAT1 and HAT2 RNAi was induced 24 h before harvest (Northern Blot data verifying knock-down of HAT and HAT2 are published elsewhere (T. Kawahara *et al.*, in preparation). All blots were stripped and reprobed with α-histone H3 (Abcam, 1791) to control for equal loading. B. Western blot analysis of H4K4 acetylation state in BF cell lysates deficient in DAC2, DAC4 or Sir2rp1.

Arguably the most direct approach to learn about the role of a histone modification is to remove the mark of interest and to look for a phenotype. To study acetylation, it is a common approach to change lysine to arginine or glutamine, to mimic deacetylated and constitutively acetylated lysine respectively (Megee *et al*., 1990). In *T. brucei*, all four core histones are encoded by tandem gene arrays (Berriman *et al*., 2005), which complicates mutagenesis. We therefore attempted to identify the enzymes that modulate the modification. In other organisms, newly synthesized histone H4 is diacetylated at K5 and K12 by a cytosolic type-B HAT1, but the function of this diacetylation is unknown (Sobel *et al*., 1995). Interestingly, no potential type-B HAT has been identified in *T. brucei* (Ivens *et al*., 2005). We therefore chose to screen all the identifiable HAT and deacetylase (DAC) homologues. The role of the non-essential HAT3, DAC2, DAC4 and Sir2rp1 genes was tested in homozygous null cell lines. All other identified histone-modifying enzymes (HAT1, HAT2, a potential HAT with a conserved PHD domain, Elp3a, Elp3b, DAC1 and DAC3) were tested in cell lines that allowed inducible RNAi-mediated depletion of the respective enzyme (Ingram and Horn, 2002; Alsford *et al*., 2007). Western blot analysis of the various cell lines indicated that HAT3 is responsible for H4K4 acetylation in BF and PF (Fig. 4A and Fig. S2). Eighty per cent of H4K4 is normally acetylated in PF *T. brucei* (Janzen *et al*., 2006b). The residual H4K4ac signal in the HAT3^–/–^ cells may be caused by the ability of one of the other HATs to inefficiently acetylate H4K4. Although we cannot exclude the possibility that our H4K4ac antibody cross-reacted at lower affinity with other sites on H4, this seems unlikely, as we did not see cross-reactivity with unmodified H4K4, H4K10ac or with any other histone (Fig. 1A). As expected, the unmodified H4K4 signal was greatly increased in the HAT3^–/–^ cell lines (Fig. 4A). Equal loading was confirmed by stripping and re-probing the membrane with an antibody (Abcam, 1791) that reacts with *T. brucei* histone H3. Several sites on the C-terminal tail of histone H2A and the N-terminal tail of histone H4 can be acetylated (Janzen *et al*., 2006b; Mandava *et al*., 2007). To assess the specificity of HAT3 for histone H4K4, we purified histones from HAT3^−/−^ cells and used Edman degradation and tandem mass spectroscopy to measure levels of acetylation on other sites known to exist in the acetylated form. Except for H4K4, no differences could be detected in the acetylation patterns of histones isolated from wild-type and HAT3^−/−^ cells (data not shown).

**Fig. 5:**
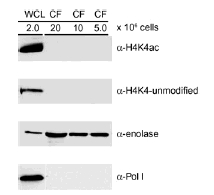
Western blot analysis of the H4K4 acetylation state of cytoplasmic histones. Whole-cell lysates (WCL) and cytoplasmic fractions (CF) from up to 2 × 10^7^ cells were analysed with α-H4K4ac, α-H4K4-unmodified, α-enolase and α-Pol 1.

Because HAT3 is found inside the nucleus (T. Kawahara *et al.*, in preparation) and unmodified H4K4 was strongly enriched in S phase, we speculated that – in contrast to other model organisms – newly synthesized histones in *T. brucei* remain unmodified at this site until they have been imported into the nucleus. To test this idea, we separated nuclear and cytosolic fractions (Rout and Field, 2001). No unmodified or acetylated H4 could be detected in the cytoplasmic fraction, suggesting that H4 is very rapidly imported into the nucleus (Fig. 5). Purity of the fractions was confirmed with antibodies specific for cytoplasmic enolase and nuclear RNA Polymerase I.

### Unmodified H4K4 decreases rapidly when protein synthesis is inhibited

None of the DAC-knockout cell lines showed a decrease in the level of unmodified H4K4 (Fig. 4B). We therefore tested the effect of RNAi-mediated depletion of the essential DAC1 and DAC3, or treatment with the HDAC inhibitor Trichostatin A (TSA). All of these treatments were accompanied by a decrease in the unmodified H4K4 signal (data not shown). Depletion of other essential enzymes also led to a decrease in unmodified H4K4 (data not shown). As one would expect for the depletion of an essential enzyme, cell growth was slowed by depletion of DAC1 or DAC3 or inhibition by TSA. The observation that any interference with an essential DAC led to a decrease in unmodified signal strongly suggested that this was an indirect effect of depleting the RNAi targets. As unmodified H4K4 is predominantly found in S phase, presumably representing newly synthesized histones, we reasoned that that loss of the unmodified H4K4 signal could be attributed to reduced cell growth and decreased histone synthesis, rather than a direct consequence of DAC depletion.

**Fig. 6:**
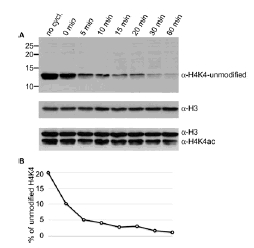
Inhibition of protein synthesis leads to a loss of unmodified H4K4 signal. A. Western blot analysis of the unmodified H4K4 signal in cells lysates from control and cycloheximide-treated cells. Time points listed refer to start of cell harvest, which included a total of ∼4 min of centrifugation before a gel-loading buffer was added and lysates were boiled. The blot was stripped and equal loading was confirmed with α-histone H3. B. Quantification of Western blot signal was based on ECL plus luminescence as measured by VersaDoc Gel Imager (Bio-Rad). Percentages are based on published data (Janzen *et al*., 2006a). Luminescence from H3 was used to normalize the H4K4-unmodified signal for differences in loading.

To test this hypothesis, we inhibited protein synthesis with cycloheximide and analysed cell lysates by Western blot over a period of 60 min (Fig. 6A). Changes in unmodified H4K4 signal were quantified (Fig. 6B), after normalization for differences in loading based on measurements with H3 antibody. H3 was used instead of H4 to avoid any complication arising from incomplete antibody stripping. Quantification revealed a remarkably rapid loss of the unmodified H4K4 signal. Previous studies have shown that untreated PF contain 20% unmodified H4K4 (Janzen *et al*., 2006b). We observed a 50% decrease to a total of 10% unmodified H4K4 when cells were harvested immediately after cycloheximide addition, meaning that cells were exposed to the inhibitor for ∼4 min during centrifugation. The unmodified H4K4 signal declined to 1.3% of total H4 after 60 min of cycloheximide treatment. Partition experiments detected no cytoplasmic accumulation of unmodified H4K4 in HAT3^−/−^ cells (Fig. S3).

## Discussion

### Cell cycle dependent regulation of H4K4 acetylation

Post-translational acetylations of histone tails have been shown to affect a large number of cellular processes, yet little is known about their function in *T. brucei*. Given the highly divergent sequence of the trypanosome N-terminal tail of histone H4, we were restrained from using commercially available antibodies. Instead, we generated new and highly specific antibodies that would allow the characterization of H4K4 in *T. brucei.*

Immunofluorescence microscopy indicated that antibodies to both unmodified H4K4 or H4K4ac cannot bind to histones in cells in G1/G0. G1-specific blockage by nearby covalent modification seemed unlikely, based on studies that failed to identify such modifications. Instead, we attributed this surprising observation to epitope masking by a non-covalently binding factor, as H4K4ac signal was clearly visible in cells in G1/G0 as shown by Western blotting. This finding suggests that epitope masking, in fixed cells that are generally used for IF analysis, can pose a serious problem when interpreting IF data. This is especially true for studies of histone modification in which antibodies are only available against the modified but not against the unmodified sites. Furthermore, our approach showed that cell cycle-specific masking of a specific epitope could be revealed by taking advantage of SDS-PAGE after cell cycle-dependent cell sorting.

We can only speculate about the nature of the factor blocking the H4K4 site. A number of acetyl-binding proteins have been described in different eukaryotes, and the classic acetyl-binding motif – the so-called bromodomain (reviewed in Winston and Allis, 1999) – appears to be conserved in some predicted proteins of *T. brucei* (Ivens *et al*., 2005).

Besides cell cycle-specific blockage of H4K4, IF analysis revealed a strong enrichment of the unmodified H4K4 residue in cells with an elongated kinetoplast. In a separate study, we showed that kinetoplast division, visualized by an elongated kinetoplast, occurs during nuclear S phase. Thus, based on our IF analysis, unmodified H4K4 is strongly enriched in S phase cells. This observation agrees with the Western blot data, which again showed a strong enrichment of unmodified H4K4 in cells in S phase.

### Is *T. brucei* H4K4 functionally equivalent to H4K5 in other organisms?

H4K4 is the most highly acetylated (80%) site identified in trypanosome histones (da Cunha *et al*., 2006; Janzen *et al*., 2006b). In this study we show that the large majority of H4K4 is acetylated by the non-essential MYST-type acetyltransferase HAT3. Thus far, no phenotype has been detected for HAT3^–/–^ in BF cultured in liquid medium (T. Kawahara *et al.*, in preparation) or grown in rodents (G.A.M. Cross, unpubl. obs.). Acetylation of H4K4 by a non-essential enzyme would be consistent with observations in other eukaryotes that H4K5 acetylation may not be essential but may be evolutionarily advantageous. Are there other similarities that would suggest a functional equivalence between H4K4 in *T. brucei* and H4K5 in other organisms? The best-studied and most conserved role of H4K5 may be in deposition of newly synthesized histones (Brownell and Allis, 1996). The majority of histone synthesis occurs during S phase (Wu and Bonner, 1981) and acetylation of H4 at K5 and K12 happens in the cytosol. This diacetylation is evolutionary conserved in flies (Sobel *et al*., 1994), humans (Sobel *et al*., 1995) and in *Tetrahymena*, where the homologous residues K4 and K11 are used (Chicoine *et al*., 1986). Diacetylation of newly synthesized H4 is thought to occur by a cytosolic HAT, traditionally defined as type-B HATs (Brownell and Allis, 1996; Parthun *et al*., 1996). The mechanistic link between cytosolic H4 acetylation and histone deposition stems from observations that the chromatin assembly factor 1 (CAF-1) selectively deposits histone H3/H4 heterodimers, acetylated in the cytoplasm, onto newly replicated DNA. Despite the conservation of these marks, evidence from yeast suggests that they are non-essential. Mutants in which all N-terminal lysines of histone H4 have been changed to arginines, to mimic a permanently deacetylated state, were viable and showed only an extended S phase (Megee *et al*., 1990). Lethality occurred only after deletion of the H3 tail in addition to substitution of H4K5, K8 and K12 with glycines (Ma *et al*., 1998). It has been shown that the H3 tail is able to substitute for the H4 tail. Thus, the function of some PTM of the H4 tail only become apparent if the H3 tail is deleted.

As in other eukaryotes, histone synthesis in *T. brucei* reaches its peak during S phase (Ersfeld *et al*., 1996; Garcia-Salcedo *et al*., 1999), but no putative cytoplasmic HATs have been identified (Ivens *et al*., 2005). In agreement with these observations, unmodified H4K4 is strongly enriched in S phase, probably representing newly synthesized histones. Unmodified H4K4 is converted to the acetylated form, suggesting that H4K4 is acetylated during or shortly after replication. It is difficult to speculate about histone deposition in *T. brucei* as many of the major components in human chromatin assembly, like the CAF-1 complex, have no obvious homologues in trypanosomes. One protein that does seem to be conserved is ASF1 (GenBank Accession No. Q4GZF6), a chaperone implicated in transport of the H3/H4 heterodimer into the nucleus. Interestingly, ASF1 binds to the core region of the H3/H4 dimer and acetylation of the tails is not necessary. Should ASF1 play a role in histone translocation in *T. brucei*, it would probably not require acetylation of the H4 tail.

Both microscopic (Taddei *et al*., 1999) and biochemical analysis (Jackson *et al*., 1976) in human cells indicate that H4 on newly assembled chromatin remains acetylated for 20–30 min before HDACs establish steady-state intermediate acetylation levels. Upon entry into mitosis and the onset of chromatin condensation, histones become hypoacetylated, which is most clearly manifested in the loss of H4K5ac (Kruhlak *et al*., 2001). Substitutions of all N-terminal lysines of histone H4 with glutamines, to mimic the hyperacetylated state, display a marked delay in progression through G2 and M phases in yeast. It has been suggested that this block is caused by a defect in chromatin condensation. Alternatively, it has been proposed that deacetylated lysine plays a role in a specific checkpoint control mechanism, as the insertion of a single additional deacetylated lysine rescues the G2/M block. Further analysis indicated that the lysine-mediated G2/M block is controlled by the RAD9-dependent DNA damage checkpoint (Megee *et al*., 1995). Cell cycle control in *T. brucei* differs significantly from other eukaryotes, and different checkpoint control mechanisms seem to be in effect (reviewed in Hammarton, 2007). No RAD9-dependent checkpoint pathway has been identified in *T. brucei*, but the observation that depletion of the non-essential DAC4 leads to a delay of G2/M phase suggests that a similar pathway may exist (Ingram and Horn, 2002).

None of the five DACs identified in *T. brucei* had a specific effect on H4K4 acetylation, and unmodified H4K4 almost disappeared soon after inhibiting protein synthesis. These observations suggest that newly synthesized histones are the only source of unmodified H4K4 and that newly synthesized histones are rapidly acetylated after import into the nucleus. Other less likely explanations for the decrease in H4K4unmodified levels would be that inhibition of protein synthesis leads to an inhibition of HDACs or to strong upregulation of HATs. We did not see a decrease in H4K4ac after S phase, not even at the onset of mitosis. *T. brucei* undergoes a closed mitosis during which no visible chromatin condensation occurs, so changes in acetylation may not be necessary from a structural point of view. The question of whether lack of chromatin condensation during mitosis is cause or consequence of constant histone acetylation may be addressed in the future in HAT3^–/–^ cells.

### Conclusions

We have shown that HAT3 is responsible for acetylation of H4K4, and our results suggest that H4K4 may not be actively deacetylated by any HDAC. It is also interesting, as our data suggest, that *T. brucei* may not diacetylate newly synthesized histones in the cytosol, in contrast to all other eukaryotes studied to date. Hyperacetylated chromatin is generally considered to be transcriptionally active and less densely packed, leaving DNA more accessible to factors that interact with DNA during transcription. The high level of H4K4 acetylation could serve to keep chromatin in an open conformation, which appears to be the general situation in *T. brucei* (Navarro *et al*., 1999), except for obvious regions of heterochromatin, mainly in the non-essential and apparently non-transcribed ‘haploid’ subtelomeric regions. The relatively small number of histone-modifying enzymes in *T. brucei* compared with humans or yeast suggests a more basic network of histone modifications in this organism. Where redundancy among modifications often masks their essential functions in other eukaryotes, histone-modifying enzymes and histone modifications in a simpler organism might have more critical and non-redundant roles. This will simplify deciphering the network of cross-talk among modifications.

## Experimental procedures

### Cell lines and culture conditions

Procyclic forms of *T. brucei* strain Lister 427 were cultured in SDM-79 (Brun and Schonenberger, 1979) supplemented with 10% fetal bovine serum and 0.25% Hemin. BFs were derived from Lister 427 MITat1.2 (clone 221a) and cultured in HMI-11 (Hirumi and Hirumi, 1989). Generation and characterization of the BF knockdown and knockout cell lines is described elsewhere (T. Kawahara *et al.* in preparation).

### Antibody generation

Polyclonal antibodies specific for unmodified or acetylated histone H4 lysine were raised by immunizing rabbits according to a 77 day protocol (Sigma) with KLH-conjugated peptides AKGKKSGEAC and AKG(Kac)KSGEAC. A general modification-independent histone H4 antibody was derived fortuitously from another rabbit immunized according to a 118 day protocol (Covance) with the KLH-conjugated peptide AKGKKSGEAC. Antisera were affinity purified using the corresponding peptides immobilized to SulfoLink coupling gels (Pierce) as described (Harlow and Lane, 1999).

### Western blot analysis and antibody characterization

A total of 2 × 10^6^ cells were lysed in RIPA buffer (50 mM Tris-HCl pH 8.0, 150 mM NaCl, 1% NP-40, 0.25% sodium-deoxycholate, 0.1% SDS), plus mammalian proteinase inhibitor cocktail (Sigma) supplemented with 200 μg ml^−1^ PMSF and 4 μg ml^−1^ pepstatin. Lysates were separated on a 15% SDS-PAGE gel and transferred onto a nitrocellulose membrane. Membranes were blocked for 1 h with 3% BSA. Antibody specificity was confirmed by ELISA and peptide competition assays. Primary antibodies were detected with horseradish-peroxidase-conjugated sheep anti-rabbit antibodies (Amersham-Pharmacia) and SuperSignal West Pico (Pierce) or ECL Plus (Amersham-Pharmacia). Intensity was quantified using a Versadoc imaging system (Bio-Rad). The antibodies to enolase and *T. brucei* POL I were gifts of N. Papavasiliou and A. Günzl respectively.

### Cell sorting

Unfixed PFs (∼1.3 × 10^7^ ml^−1^) were incubated at room temperature in phosphate-buffered saline (PBS) for 30 min in 5 μM DyeCyclin Orange (Invitrogen). To stain after fixation, 2 × 10^7^ cells were washed twice with PBS, re-suspended in 200 μl of PBS and fixed by addition of 2 ml of ice-cold 70% ethanol while vortexing. Cells were stored overnight at 4°C, pelleted and re-suspended in 0.5 ml of PBS containing 2 mM EDTA, 200 μg ml^−1^ RNAseA, 2.5 μg ml^−1^ Propidium iodide and incubated at 37°C for 30 min. Cell sorting, based on relative DNA content, was performed in the Rockefeller University Flow Cytometry Resource Center using a FACSAria (BD Biosciences).

### Immunofluorescence

Cells were suspended at 1 × 10^7^ ml^−1^ in SDM-79 containing 2% formaldehyde for 5 min at room temperature and washed twice with PBS. The fixed cells were allowed to settle onto aminopropyltriethoxysilane-coated coverslips and permeabilized by immersion for 5 min in PBS containing 0.1% NP-40. After blocking by two rinses of 10 min with PBG (PBS containing 0.2% cold fish gelatin (Sigma) and 0.5% (BSA), the coverslips were incubated with primary antibody for 1 h. Subsequently, the cells were washed by four rinses of 5 min with PBG and incubated with the corresponding secondary antibody for 1 h then stained with DAPI (1.0 ng μl^−1^) for 10 min and mounted in antifade mounting solution (Vectashield, Vecta Laboratories). Vertical stacks of 15–25 slices (0.2 μm steps) were captured using a DeltaVision microscope (Applied Precision). Deconvolution and pseudo-colouring was performed using softWoRxTM v3.5.1 software. Subsequently, 7–15 images from vertical stacks were merged. The antibody to tubulin was a gift from Keith Gull.

## Supplementary material

This material is available as part of the online article from: http://www.blackwell-synergy.com/doi/abs/10.1111/j.1365-2958.2007.06079.x

(This link will take you to the article abstract).
