# Supplementary material for: Acetylation of histone H4K4 is cell cycle regulated and mediated by HAT3 in Trypanosoma brucei
Source: Mol Microbiol. 2008 Feb;67(4):762–71. doi: 10.1111/j.1365-2958.2007.06079.x (PMC2253726; doi:10.1111/j.1365-2958.2007.06079.x)
Supplement: Supplementary file 1 [file mmi0067-0762-SD1.pdf]

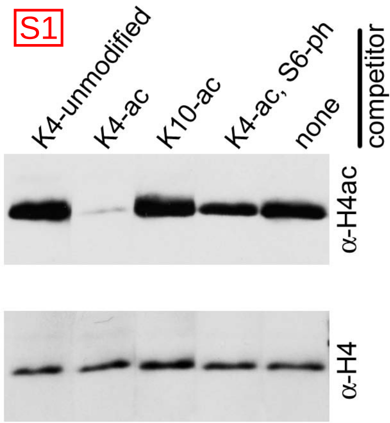
S1
K4-unmodified
K4-ac
K10-ac
K4-ac, S6-ph
none
competitor
α-H4ac
α-H4

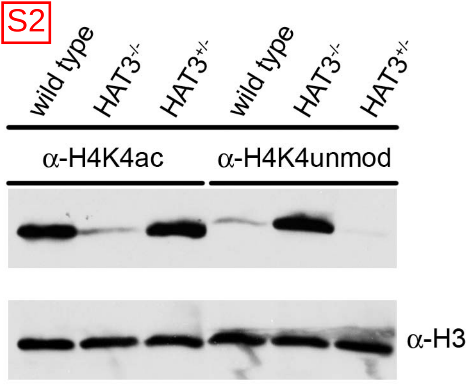
S2
wild type
HAT3-/-
HAT3+/-
wild type
HAT3-/-
HAT3+/-
α-H4K4ac
α-H4K4unmod
α-H3

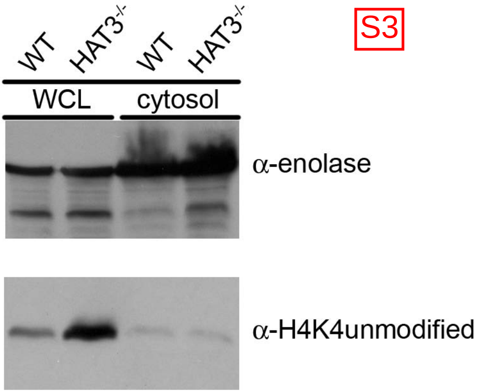
S3
WT
HAT3-/-
WT
HAT3-/-
WCL
cytosol
α-enolase
α-H4K4unmodified

LEGENDS

Figure S1: HAT3 acetylates H4K4 in PF. Western blot analysis of the H4K4 acetylation state in PF cell lysates.

Figure S2: Characterization of anti-H4ac. Western blot of whole trypanosome extracts ($2x10^6$ cells/lane). Peptide competitors used are shown above each lane. To confirm equal loading, blots were stripped and reprobed with anti-H4 in the absence of peptide competitors.

Figure S3: Western blot analysis of the H4K4 acetylation state of cytoplasmic histones. Whole cell lysates (WCL) and cytoplasmic fractions (cytosol) from 5×10^6 and 2.75×10^7 cells respectively, were analyzed with antibodies to unmodified H4HK4 and enolase.
